# Deep neural networks for wearable sensor-based activity recognition in Parkinson’s disease: investigating generalizability and model complexity

**DOI:** 10.1186/s12938-024-01214-2

**Published:** 2024-02-09

**Authors:** Shelly Davidashvilly, Maria Cardei, Murtadha Hssayeni, Christopher Chi, Behnaz Ghoraani

**Affiliations:** 1 Electrical and Computer Engineering, Florida Atlantic University, Boca Raton, FL 33431 US; 2 Biomedical Engineering, University of Florida, Gainesville, FL US; 3 Computer Engineering, University of Technology, Baghdad, Iraq

**Keywords:** Wearable sensors, Human activity recognition, Parkinson’s disease, Deep neural networks, Generalizability, Data augmentation, Domain adaptation

## Abstract

**Background:**

The research gap addressed in this study is the applicability of deep neural network (NN) models on wearable sensor data to recognize different activities performed by patients with Parkinson’s Disease (PwPD) and the generalizability of these models to PwPD using labeled healthy data.

**Methods:**

The experiments were carried out utilizing three datasets containing wearable motion sensor readings on common activities of daily living. The collected readings were from two accelerometer sensors. PAMAP2 and MHEALTH are publicly available datasets collected from 10 and 9 healthy, young subjects, respectively. A private dataset of a similar nature collected from 14 PwPD patients was utilized as well. Deep NN models were implemented with varying levels of complexity to investigate the impact of data augmentation, manual axis reorientation, model complexity, and domain adaptation on activity recognition performance.

**Results:**

A moderately complex model trained on the augmented PAMAP2 dataset and adapted to the Parkinson domain using domain adaptation achieved the best activity recognition performance with an accuracy of 73.02%, which was significantly higher than the accuracy of 63% reported in previous studies. The model’s F1 score of 49.79% significantly improved compared to the best cross-testing of 33.66% F1 score with only data augmentation and 2.88% F1 score without data augmentation or domain adaptation.

**Conclusion:**

These findings suggest that deep NN models originating on healthy data have the potential to recognize activities performed by PwPD accurately and that data augmentation and domain adaptation can improve the generalizability of models in the healthy-to-PwPD transfer scenario. The simple/moderately complex architectures tested in this study could generalize better to the PwPD domain when trained on a healthy dataset compared to the most complex architectures used. The findings of this study could contribute to the development of accurate wearable-based activity monitoring solutions for PwPD, improving clinical decision-making and patient outcomes based on patient activity levels.

**Supplementary Information:**

The online version contains supplementary material available at 10.1186/s12938-024-01214-2.

## Background

Human activity recognition (HAR) with wearable sensors has gained immense popularity due to its promising applications in numerous fields, such as health monitoring, sports performance analysis, and patient rehabilitation [1]. Utilizing powerful, deep neural network (NN) models in HAR has significantly improved the accuracy and robustness of activity recognition systems [2]. However, the generalizability of deep NN models remains a significant challenge, especially when dealing with small datasets or data from a different domain. This limitation is particularly relevant for Parkinson’s disease (PD) patients, whose disease symptoms affect movement patterns, making it challenging to generalize results obtained from healthy populations. PD is a neurodegenerative disorder that affects movement patterns and is characterized by tremors, rigidity, and bradykinesia.

The need to develop accurate and robust deep NN models for HAR in patients with PD (PwPD) is prevalent. By having access to accurate activity recognition data, physicians can gain insights into a patient’s daily routines and physical behaviors. This knowledge allows them to fine-tune their therapeutic approaches to align with each patient’s unique activity patterns and lifestyle. Such personalized adjustments can be instrumental in achieving optimal treatment efficacy, leading to enhanced patient outcomes and improved quality of life [3]. In addition, these tools offer patients the means to autonomously monitor and understand the relationship between their physical activity levels and the intensity of their symptoms. This understanding, in turn, paves the way for informed lifestyle modifications, enabling better symptom management.

Wearable sensor data from the healthy population are easier to collect in larger quantities with better data quality and are more readily available to the public. However, collecting labeled data from PwPD at different stages of the disease during different activities of daily living can be costly and burdensome. This often leads to insufficient quantities of labeled data, making it challenging to develop accurate and generalizable models. The application of wearable sensors and deep NN models for human activity recognition has been widely investigated in the literature. However, most studies focus on healthy individuals or specific disease populations, such as stroke patients or elderly individuals [1, 2].

Limited research has been conducted on developing accurate classification models for activity recognition in the context of a healthy-to-disease domain shift, particularly in the case of PD. The only study similar to our work that explored cross-testing of healthy-to-PwPD data is the work of Albert et al. [4], which utilized smartphone accelerometer data and traditional machine learning models to classify healthy and PD activity data across 9 activities. However, they observed a significant reduction in performance with only 63.5% accuracy when directly applying the same models to PD data, with an improvement observed after utilizing PD data in training—highlighting the need for tailored tools and analyses for specific patient populations. Two other papers related to the present paper are Jalloul et al. [5] and Som et al. [6]. Jalloul et al. developed a k-nearest neighbors model using data on 7 activities from healthy individuals and only one PwPD, achieving higher classification accuracy for healthy individuals and only 44.3% accuracy for the PwPD. This highlights the need for further adaptation when applying activity classification to PwPD. Som et al. [6] developed a binary classifier to classify walking vs. non-walking using a healthy source dataset and a target dataset mixed with 16 healthy and 18 PwPD data. They showed the feasibility of leveraging healthy data to develop classifiers for the PwPD with maximum accuracy and F1 score of 73.81%. However, their target domain contained healthy data. The present study differs from previous works as we present the first investigation of the state-of-the-art deep NN-based methods with raw data for applying healthy-to-PwPD human activity recognition.

Data augmentation and transfer learning have been the focus of several studies to enhance classification accuracy in applications other than activity recognition for PwPD. For example, Kalouris et al. [7] applied rotation, jitter, scaling, and permutation data augmentation methods on a with older adults performing 6 activities and used two publicly available healthy datasets—PAMAP2 and UCI-HAR—as the source domains. They achieved a maximum accuracy of 84.89% utilizing a supervised, homogeneous, divergence-based domain adaptation method with various convolutional neural networks (CNNs) as fixed feature extractors for the target dataset containing 6 activities. They found that the rotation and permutation methods were the most successful when transferring across domains. In another study, Um et al. [8] tested the same augmentation techniques as Kalouris et al. [7] to detect medication states in Parkinson’s patients and found that the best-performing augmentation methods alleviated sensor position variability and event locations in an arbitrarily segmented window.

Domain adaptation strategies, such as the state-of-the-art Domain Adversarial Neural Network (DANN) proposed by Ganin et al. [9], have outperformed other techniques in the image classification task on the MNIST and Office datasets. However, there are few investigations into the advantages of such domain adaptation strategies to enhance generalizability from healthy to diseased populations, particularly using wearable data. The closest studies that utilize domain adaptation techniques for cross-domain activity classification are the works of Chen et al. [10] and Hosseini et al. [11], but not for the specific application of healthy-to-PwPD cross-testing for activity recognition. Chen et al. [10] proposed a technique called Stratified Transfer Learning for domain adaptation, which involved source domain selection and activity transfer to capture domain-specific properties. They conducted similar work by classifying activities from the healthy-subject data in PAMAP and MHEALTH and cross-testing between datasets. Their technique achieved an F1 score of 55% when testing with chest data from MHEALTH as the source to chest data from PAMAP as the target, with F1 scores ranging from 39 to 59% for other cross-testing cases. Hosseini et al. [11] employed a Bidirectional LSTM Recurrent Neural Network with Maximum Mean Discrepancy loss to reduce confusion between the source and target domains. Their objective was similar to the current study as they utilized adult HAR data as source data to predict activities for a children’s activity dataset. They reported binary F1 scores for each of the 5 activities reported, with a maximum score of approximately 70% for standing, 63% for walking, and 52% for sitting. However, none of the previous studies investigated using data augmentation techniques and domain adaptation for activity recognition applications in PwPD data.

This paper addresses the challenge of generalizing deep NN models trained on young, healthy populations to older PD patients for healthy-to-PwPD cross-domain activity recognition. The experiments uniquely focus on exploring model complexity alongside data augmentation, manual sensor axes reorientation, and domain adaptation methods to improve accuracy and robustness across healthy-to-PwPD domains. The study expands upon the authors’ preliminary work [12] and provides this comprehensive investigation into the generalizability of deep NN models in this specific scenario to help guide future research in this field.

## Results

**Fig. 1:**
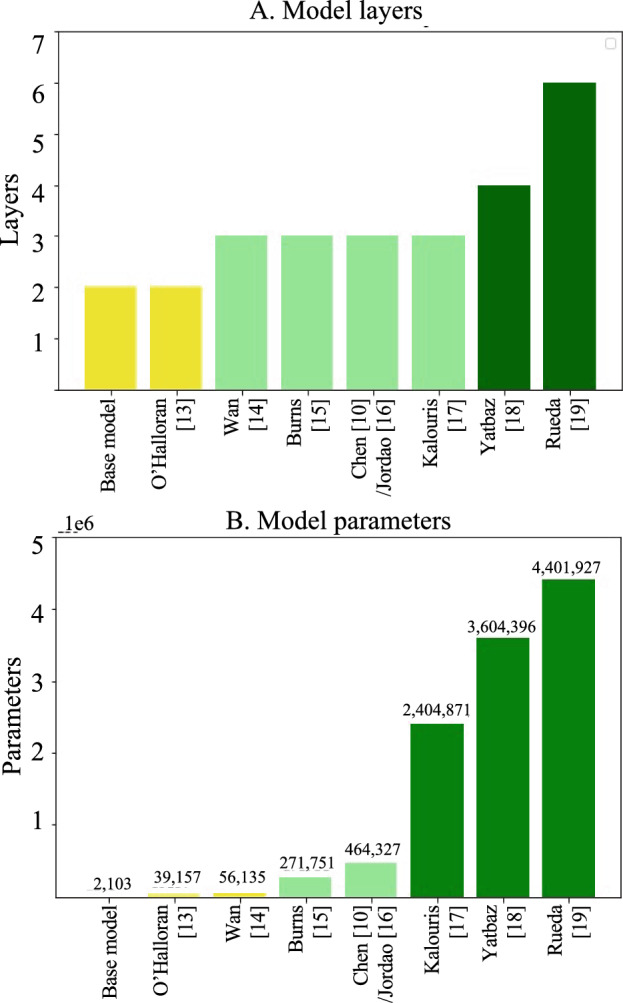
The figure compares the number of layers (**A**) and parameters (**B**) in the implemented CNN architectures

The study utilizes CNN models from the HAR literature [10, 13–19] that vary in complexity in terms of the number of layers and parameters, as well as a base CNN model designed with a more simple architecture compared to the other models. Figure 1 compares the number of layers and parameters between the models implemented in the work. The models were trained in a k-fold cross-validation, holding out each subject’s data for testing. The best model from each cross-validation was loaded and cross-tested directly against data of a different domain, both in healthy-to-healthy and healthy-to-PwPD test cases. Including the healthy-to-healthy test cases was intended to serve as a baseline comparison for results and justification for methods used for the healthy-to-PwPD test case. Any degradation of accuracy values in the healthy-to-PwPD scenario compared to the healthy-to-healthy test case will be directly quantified. The effects of data augmentation, manual sensor axes reorientation, and deep domain adaptation were explored in the cross-testing scenarios. The data augmentation methods utilized in the study aimed to accomplish two major goals—namely, simulating different sensor placements and mimicking sensor noise due to Parkinson’s motor complications. In practice, 3D wearable body sensors can exhibit different orientations of their X, Y, and Z axes depending on the design of the actual sensor itself and the placement of the sensor on the body. This can introduce significant variability in activity motion data that decreases model generalizability. Therefore, in addition to data augmentation on the training samples, we manually reoriented the sensor axes between datasets to match the data samples better. Last, we utilized a state-of-the-art domain adaptation technique—Domain Adversarial Neural Network (DANN)—in another set of tests to learn discriminative and domain invariant features between domains. All three aforementioned methods were incorporated to increase the models’ ability to generalize to the PwPD domain, considering they were all trained on healthy data.

Two publicly available healthy datasets, MHEALTH [20] and PAMAP2 [21], were utilized for the study, alongside a private Parkinson’s disease dataset referred to as PD data throughout the paper. Each dataset consisted of subjects performing various activities of daily living while wearing IMU sensors. Wrist and ankle sensor data were utilized in this study. For PAMAP2, the sensors were affixed to the subject’s dominant-side body. For MHEALTH, the sensors were placed on the right wrist and left ankle. For the PD dataset, sensors were placed on the patient’s most affected side. All the data were preprocessed to maintain similar activities, units, and window segmentation across datasets. The ’Methods’ section outlines further details regarding the datasets and data preprocessing steps.

The study used different performance metrics depending on the cross-testing type. In the case of healthy-to-healthy cross-testing, the performance metric used was the accuracy between the actual and predicted labels, as both datasets were balanced. F1 scores were also reported for completion. However, for the healthy-to-PwPD cross-testing, average binary accuracy and F1 scores were used to evaluate the models’ performance. This decision was made due to the imbalanced nature of the PD dataset, which required taking both true positives and false negatives into account [22]. In addition, when it came to domain adaptation for healthy-to-PwPD cross-testing, our analyses focused on the average binary F1 scores, as the average binary accuracy did not provide much insight into the performance of the models. Using different performance metrics for different types of cross-testing provided a more comprehensive evaluation of the effectiveness of the considered deep NN models.

### Cross-validation

The first set of tests involved leave-one-subject-out cross-validation, where each architecture was tested on a single subject while trained on all the remaining subjects. The model parameters were tuned using 80% and 20% subject splits during training. Table 1 presents the average testing accuracy values across eightfold for the subjects in PAMAP2 and tenfold for the subjects in MHEALTH. The architectures are ordered based on the number of convolutional layers, from smallest to largest. The highest average accuracy for the original MHEALTH data was 94.57% using the Kalouris architecture, which decreased slightly to 91.92% with data augmentation. Among the augmented MHEALTH data, the Burns architecture had the highest average accuracy at 94.98%, a 4.49% increase in average accuracy from the original data. For PAMAP2, the base model achieved the highest average accuracy on the original data at 83.98%, which decreased to 80.74% with data augmentation. The highest data augmentation average accuracy was 89.91% with the Burns model, which was a 13.19% increase from the original PAMAP2 data.

**Table 1:** The table presents the average cross-validation accuracy results for different models, where ’MH Orig’ refers to the original MHEALTH data, ’MH Aug’ refers to the augmented MHEALTH data, and the same notation applies to PAMAP2

	Base Model	O’Halloran [13]	Wan [14]	Burns [15]	Chen/Jordao [10, 16]	Kalouris [17]	Yatbaz [18]	Rueda [19]
MH Orig	$$\begin{array}{l}91.70\\ (91.30)\end{array}$$	$$\begin{array}{l}88.15\\ (87.73)\end{array}$$	$$\begin{array}{l}93.36\\ (96.13)\end{array}$$	$$\begin{array}{l}90.49\\ (89.72)\end{array}$$	$$\begin{array}{l}58.11\\ (54.79)\end{array}$$	$$\begin{array}{l}94.57\\ (94.36)\end{array}$$	$$\begin{array}{l}81.06\\ (80.83)\end{array}$$	$$\begin{array}{l}87.21\\ (86.35)\end{array}$$
MH Aug	$$\begin{array}{l}86.72\\ (85.73)\end{array}$$	$$\begin{array}{l}92.04\\ (91.64)\end{array}$$	$$\begin{array}{l}93.51\\ (93.52)\end{array}$$	$$\begin{array}{l}94.98\\ (94.95)\end{array}$$	$$\begin{array}{l}84.19\\ (83.36)\end{array}$$	$$\begin{array}{l}91.92\\ (91.91)\end{array}$$	$$\begin{array}{l}90.34\\ (90.01)\end{array}$$	$$\begin{array}{l}91.77\\ (91.68)\end{array}$$
P2 Orig	$$\begin{array}{l}83.98\\ (84.06)\end{array}$$	$$\begin{array}{l}82.74\\ (82.57)\end{array}$$	$$\begin{array}{l}80.74\\ (80.89)\end{array}$$	$$\begin{array}{l}76.72\\ (76.47)\end{array}$$	$$\begin{array}{l}69.03\\ (67.38)\end{array}$$	$$\begin{array}{l}81.09\\ (81.32)\end{array}$$	$$\begin{array}{l}79.69\\ (79.57)\end{array}$$	$$\begin{array}{l}83.01\\ (82.96)\end{array}$$
P2 Aug	$$\begin{array}{l}80.74\\ (78.50)\end{array}$$	$$\begin{array}{l}86.61\\ (86.57)\end{array}$$	$$\begin{array}{l}82.21\\ (87.26)\end{array}$$	$$\begin{array}{l}89.91\\ (89.86)\end{array}$$	$$\begin{array}{l}80.82\\ (77.84)\end{array}$$	$$\begin{array}{l}88.10\\ (88.17)\end{array}$$	$$\begin{array}{l}85.39\\ (85.34)\end{array}$$	$$\begin{array}{l}87.34\\ (87.22)\end{array}$$

The average values are shown, with F1 scores presented in parentheses

### Cross-testing

Direct cross-testing was conducted between domains in healthy-to-healthy and healthy-to-PwPD test cases in the second set of experiments. When testing the models on the PD dataset, the predicted labels were binarized to evaluate each activity individually. For instance, to assess the model’s performance on walking, the predicted labels for cycling and running were labeled as non-walking, while predicted walking labels were retained as walking. The direct cross-testing analysis provides insights into the models’ effectiveness in addressing domain shifts in human activity recognition. Hence, the best-performing fold model from the source dataset’s cross-validation was loaded and applied directly to the target dataset, and the mean and standard deviation used to normalize the source data were also applied to the target data. The accuracy of the model predictions in the healthy-to-healthy test cases, with PAMAP2 and MHEALTH as the source datasets, is shown in Tables 2 and 3, respectively. These tables also show the effects of data augmentation, axis reorientation, and model layer complexity on the performance. Across all architectures and datasets, manual axes reorientation significantly increased accuracy in the source data’s original and augmented test cases. The combination of data augmentation and manual reorientation in the source and target datasets had the most successful direct cross-testing performance. In this test case, the base model had the best performance of 87.43% with PAMAP2 as the source, and Burns architecture had the best performance of 76.15% with MHEALTH as the source. These models were among the simpler architectures in terms of layers and parameters. Based on these trends, it was decided to reorient the PD data to match the source data in all healthy-to-PwPD test cases.

**Table 2:** The PAMAP2 to MHEALTH direct cross-testing accuracy results

	Base Model	O’Halloran [13]	Wan [14]	Burns [15]	Chen/Jordao [10, 16]	Kalouris [17]	Yatbaz [18]	Rueda [19]
$$\begin{array}{l}\hbox {Source: P2 Orig} \\ \hbox {Target: MH Orig}\end{array}$$	$$\begin{array}{l}3.14\\ (0.95)\end{array}$$	$$\begin{array}{l}7.28\\ (7.15)\end{array}$$	$$\begin{array}{l}3.71\\ (1.84)\end{array}$$	$$\begin{array}{l}2.93\\ (0.85)\end{array}$$	$$\begin{array}{l}12.87\\ (9.46)\end{array}$$	$$\begin{array}{l}5.85\\ (7.11)\end{array}$$	$$\begin{array}{l}5.85\\ (6.37)\end{array}$$	$$\begin{array}{l}9.71\\ (12.27)\end{array}$$
$$\begin{array}{l}\hbox {Source: P2 Orig} \\ \hbox {Target: MH Reorient}\end{array}$$	$$\begin{array}{l}48.16\\ (43.95)\end{array}$$	$$\begin{array}{l}35.75\\ (34.33)\end{array}$$	$$\begin{array}{l}38.41\\ (36.49)\end{array}$$	$$\begin{array}{l}36.83\\ (35.18)\end{array}$$	$$\begin{array}{l}36.46\\ (33.61)\end{array}$$	$$\begin{array}{l}29.97\\ (27.57)\end{array}$$	$$\begin{array}{l}53.00\\ (50.41)\end{array}$$	$$\begin{array}{l}34.02\\ (25.86)\end{array}$$
$$\begin{array}{l}\hbox {Source: P2 Aug}\\ \hbox {Target: MH Orig} \end{array}$$	$$\begin{array}{l}43.74\\ (41.76)\end{array}$$	$$\begin{array}{l}54.43\\ (52.38)\end{array}$$	$$\begin{array}{l}37.96\\ (40.79)\end{array}$$	$$\begin{array}{l}44.05\\ (43.79)\end{array}$$	$$\begin{array}{l}32.48\\ (30.56)\end{array}$$	$$\begin{array}{l}55.03\\ (53.46)\end{array}$$	$$\begin{array}{l}57.31\\ (53.50)\end{array}$$	$$\begin{array}{l}41.90\\ (42.88)\end{array}$$
$$\begin{array}{l}\hbox {Source: P2 Aug}\\ \hbox { Target: MH Reorient}\end{array}$$	$$\begin{array}{l}87.43\\ (87.33)\end{array}$$	$$\begin{array}{l}73.52\\ (73.29)\end{array}$$	$$\begin{array}{l}72.36\\ (71.27)\end{array}$$	$$\begin{array}{l}77.01\\ (76.12)\end{array}$$	$$\begin{array}{l}68.49\\ (63.91)\end{array}$$	$$\begin{array}{l}74.01\\ (72.86)\end{array}$$	$$\begin{array}{l}82.63\\ (81.67)\end{array}$$	$$\begin{array}{l}79.07\\ (79.16)\end{array}$$

‘MH Reorient’ refers to MHEALTH with the sensor axes reoriented to match PAMAP2 (the source), and ‘P2 Aug’ refers to augmented PAMAP2 data. The values are shown, with F1 scores presented in parentheses

**Table 3:** The MHEALTH to PAMAP2 direct cross-testing accuracy results

	Base Model	O’Halloran [13]	Wan [14]	Burns [15]	Chen/Jordao [10, 16]	Kalouris [17]	Yatbaz [18]	Rueda [19]
$$\begin{array}{l}\hbox {Source: MH Orig} \\ \hbox {Target: P2 Orig}\end{array}$$	$$\begin{array}{l}3.49\\ (2.45)\end{array}$$	$$\begin{array}{l}14.67\\ (5.94)\end{array}$$	$$\begin{array}{l}12.63 \\ (7.79)\end{array}$$	$$\begin{array}{l}24.73\\ (13.57)\end{array}$$	$$\begin{array}{l}6.40\\ (3.52)\end{array}$$	$$\begin{array}{l}14.88\\ (10.76)\end{array}$$	$$\begin{array}{l}15.36\\ (10.79)\end{array}$$	$$\begin{array}{l}5.50\\ (3.75)\end{array}$$
$$\begin{array}{l}\hbox {Source: MH Orig} \\ \hbox {Target: P2 Reorient}\end{array}$$	$$\begin{array}{l}57.90\\ (55.23)\end{array}$$	$$\begin{array}{l}57.28\\ (53.65)\end{array}$$	$$\begin{array}{l}61.95\\ (59.59)\end{array}$$	$$\begin{array}{l}53.77\\ (50.83)\end{array}$$	$$\begin{array}{l}37.38\\ (28.90)\end{array}$$	$$\begin{array}{l}58.75\\ (57.62)\end{array}$$	$$\begin{array}{l}43.71\\ (43.61)\end{array}$$	$$\begin{array}{l}67.91\\ (65.28)\end{array}$$
$$\begin{array}{l}\hbox {Source: MH Aug} \\ \hbox {Target: P2 Orig}\end{array}$$	$$\begin{array}{l}43.29\\ (40.86)\end{array}$$	$$\begin{array}{l}52.88\\ (48.75)\end{array}$$	$$\begin{array}{l}42.50\\ (39.67)\end{array}$$	$$\begin{array}{l}47.42\\ (44.27)\end{array}$$	$$\begin{array}{l}24.86\\ (22.09)\end{array}$$	$$\begin{array}{l}47.89\\ (44.86)\end{array}$$	$$\begin{array}{l}43.35\\ (40.68)\end{array}$$	$$\begin{array}{l}47.49\\ (43.51)\end{array}$$
$$\begin{array}{l}\hbox {Source: MH Aug} \\ \hbox {Target: P2 Reorient}\end{array}$$	$$\begin{array}{l}66.74\\ (64.58)\end{array}$$	$$\begin{array}{l}63.42\\ (59.84)\end{array}$$	$$\begin{array}{l}66.27\\ (63.83)\end{array}$$	$$\begin{array}{l}76.15\\ (75.96)\end{array}$$	$$\begin{array}{l}64.08\\ (60.94)\end{array}$$	$$\begin{array}{l}71.84\\ (70.12)\end{array}$$	$$\begin{array}{l}65.56\\ (63.40)\end{array}$$	$$\begin{array}{l}69.65\\ (67.68)\end{array}$$

‘P2 Reorient’ refers to PAMAP2 with the sensor axes reoriented to match MHEALTH (the source), and ‘MH Aug’ refers to augmented MHEALTH data. The values are shown, with F1 scores presented in parentheses

The next experiment assessed how well the models trained on healthy data, including data augmentation and manual reorientation, could predict the PD dataset’s walking, sitting, and standing activities. Table 4 shows the average binary accuracy and F1 scores of predictions on PwPD activities, with MHEALTH and PAMAP2-trained models as the source. Both datasets use the same notation for data augmentation, as previously mentioned. However, the accuracy values do not represent the results due to the large class imbalance in the PD dataset. The PD dataset includes 654 walking samples, 2843 sitting samples, and 3042 standing samples. Therefore, the average binary F1 scores for each PD activity are reported in Table 4 to represent the results better. From the F1 scores in Table 4, it can be observed that data augmentation plays a significant role when cross-testing from a healthy dataset to a PD dataset. The first two rows mostly indicate a 0% F1 score without data augmentation. However, with data augmentation, the F1 score significantly increased for 15 of the 16 trials, with a 33.84% increase in the case using the O’Halloran model with augmented MHEALTH data as the source and reoriented PD as the target. This leads to the highest F1 score when MHEALTH is the source, being 36.72% (O’Halloran model), and 33.66% when PAMAP2 is the source (the base model).

**Table 4:** The binary accuracy and F1 scores for cross-testing single and combined source datasets on the PD dataset

	Base Model	O’Halloran [13]	Wan [14]	Burns [15]	Chen/Jordao [10, 16]	Kalouris [17]	Yatbaz [18]	Rueda [19]
$$\begin{array}{l}\hbox {Source: P2 Orig} \\ \hbox {Target: PD Reorient} \end{array}$$	$$\begin{array}{l}66.67\\ (0)\end{array}$$	$$\begin{array}{l}66.67\\ (0)\end{array}$$	$$\begin{array}{l}66.67\\ (0)\end{array}$$	$$\begin{array}{l}66.67\\ (0)\end{array}$$	$$\begin{array}{l}66.67\\ (0)\end{array}$$	$$\begin{array}{l}66.67\\ (0)\end{array}$$	$$\begin{array}{l}66.67\\ (0)\end{array}$$	$$\begin{array}{l}66.67\\ (0)\end{array}$$
$$\begin{array}{l}\hbox {Source: MH Orig} \\ \hbox {Target: PD Reorient} \end{array}$$	$$\begin{array}{l}66.67\\ (0)\end{array}$$	$$\begin{array}{l}62.38\\ (2.88)\end{array}$$	$$\begin{array}{l}66.67\\ (0)\end{array}$$	$$\begin{array}{l}66.67\\ (0)\end{array}$$	$$\begin{array}{l}66.67\\ (0)\end{array}$$	$$\begin{array}{l}66.67\\ (0)\end{array}$$	$$\begin{array}{l}66.67\\ (0)\end{array}$$	$$\begin{array}{l}66.67\\ (0)\end{array}$$
$$\begin{array}{l}\hbox {Source: P2 Aug} \\ \hbox {Target: PD Reorient}\end{array}$$	$$\begin{array}{l}65.23\\ (33.66)\end{array}$$	$$\begin{array}{l}62.86\\ (26.27)\end{array}$$	$$\begin{array}{l}64.32\\ (28.63)\end{array}$$	$$\begin{array}{l}66.46\\ (0)\end{array}$$	$$\begin{array}{l}66.07\\ (16.50)\end{array}$$	$$\begin{array}{l}63.89\\ (22.48)\end{array}$$	$$\begin{array}{l}65.78\\ (17.81)\end{array}$$	$$\begin{array}{l}61.26\\ (17.82)\end{array}$$
$$\begin{array}{l}\hbox {Source: MH Aug} \\ \hbox {Target: PD Reorient}\end{array}$$	$$\begin{array}{l}72.18\\ (30.78)\end{array}$$	$$\begin{array}{l}70.64\\ (36.72)\end{array}$$	$$\begin{array}{l}71.17\\ (35.55)\end{array}$$	$$\begin{array}{l}66.01\\ (19.14)\end{array}$$	$$\begin{array}{l}67.45\\ (11.82)\end{array}$$	$$\begin{array}{l}64.34\\ (28.15)\end{array}$$	$$\begin{array}{l}68.53\\ (25.81)\end{array}$$	$$\begin{array}{l}68.63\\ (34.66)\end{array}$$
$$\begin{array}{l}\hbox {Source: P2 Aug AND} \\ \hbox {MH Reorient Aug} \\ \hbox {Target: PD Reorient}\end{array}$$	$$\begin{array}{l}55.56\\ (35.80)\end{array}$$	$$\begin{array}{l}71.25\\ (44.96)\end{array}$$	$$\begin{array}{l}73.74\\ (49.04)\end{array}$$	$$\begin{array}{l}66.37\\ (1.30)\end{array}$$	$$\begin{array}{l}63.85\\ (16.51)\end{array}$$	$$\begin{array}{l}66.67\\ (0)\end{array}$$	$$\begin{array}{l}66.67\\ (0)\end{array}$$	$$\begin{array}{l}67.37\\ (25.13)\end{array}$$

The average values are shown, with F1 scores presented in parentheses. The notation for data augmentation applies to both the MHEALTH and PAMAP2 datasets. ‘PD Reorient’ indicates PD data with sensor axes reoriented to match the source data, or to PAMAP2 in the case of the combined source datasets

**Fig. 2:**
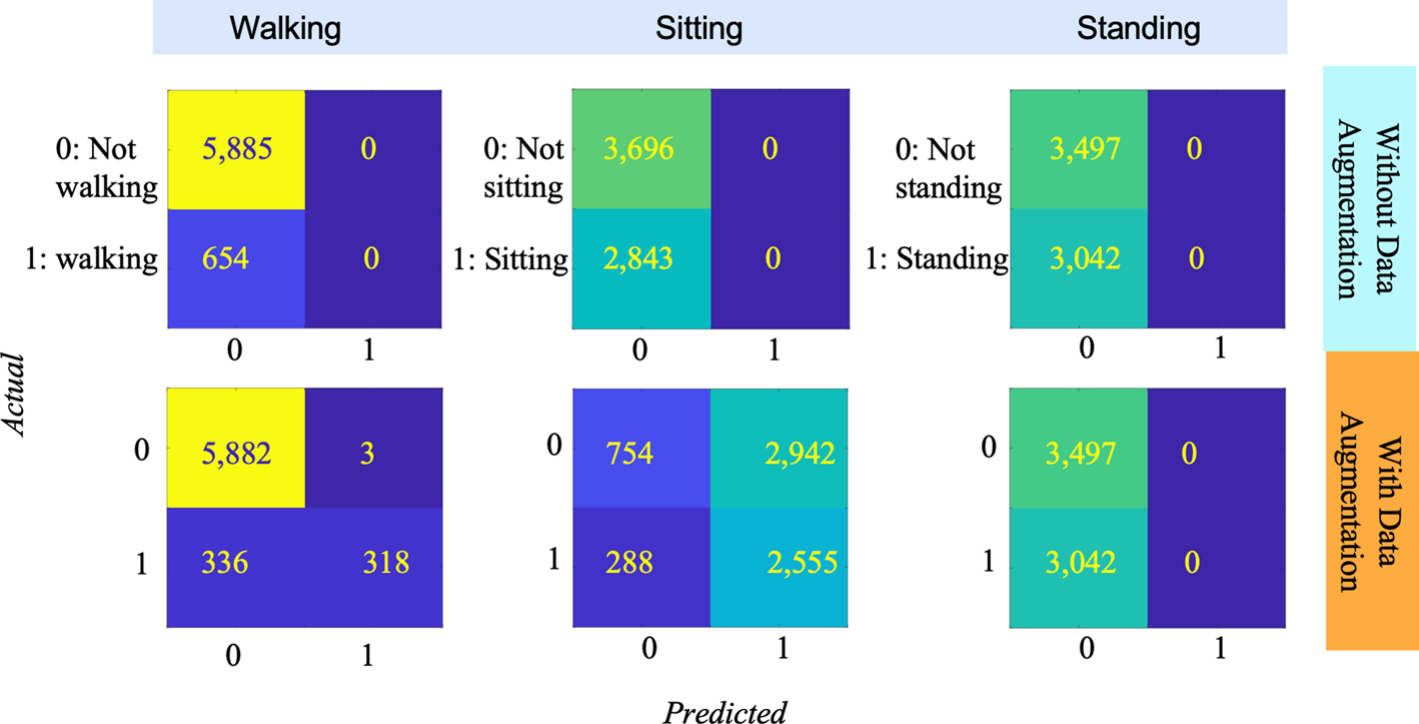
Confusion matrices for base model in PAMAP2-to-PwPD test case before and after data augmentation

To further visualize and analyze the base model’s performance before and after data augmentation in the PAMAP2-to-PwPD test case, the confusion matrices are reported in Fig. 2. These matrices show how the model classified each activity compared to one another as a binary prediction, such as ’walking’ vs. ’not walking.’ The results indicate that the models tended to largely or completely misclassify entire groups of activities. However, data augmentation alleviated this issue, enabling the models to differentiate between activities better and obtain a higher F1 score. Despite employing techniques such as regularization, early stopping, and the strategic use of validation sets to prevent overfitting, the method without data augmentation struggled to detect walking episodes. This challenge is likely attributed to the distinct walking patterns exhibited by Parkinson’s patients, especially those in advanced stages, which can markedly differ from those of healthy individuals. Given that our dataset predominantly features PwPD in their advanced stages, with an average disease duration of 10 ± 4 years, this presented significant challenges in accurately distinguishing traditional walking patterns.

To further verify our conclusions, we conducted an experiment combining the healthy datasets (MHEALTH and PAMAP2) into one dataset. Since it has been observed that a larger number of training data results in better model performance, we ran an experiment to test whether combining PAMAP2 and MHEALTH into one large training dataset would improve model performance for predicting the activity of the PD dataset. For these experiments, we combined augmented PAMAP2 with augmented MHEALTH, which was reoriented to PAMAP2 sensor axes for consistency. Augmented datasets were combined because models performed better with augmented training data in our prior experiments. We reoriented the PD dataset to PAMAP2 axes and used it as the testing dataset for the model. The results of these experiments can be found in the fifth row of Table 4. The results when using combined MHEALTH and PAMAP2 datasets for training were better than using a single source dataset in most cases, particularly for the models with simpler architectures. When the datasets are combined, the highest F1 score is 49.04% (Wan model). Similar to the experiments where we used a single dataset as the source/training dataset, it can be observed that the highest F1 scores occur with the simplest architectures tested, suggesting that decreased model complexity can help generalize between domains. In addition, combining training datasets improved the evaluation metrics in most cases, suggesting that this can be a valuable technique to investigate further.

We performed another experiment to examine how the accuracy of activity recognition models for PD patients is affected by the amount of healthy subject data used in training. This investigation aimed to determine if increasing the size of the source dataset would improve the model’s ability to generalize from healthy to PD subjects, considering their distinct movement patterns. Our findings indicated that incorporating more healthy subject data into the training set generally led to higher accuracy in classifying PD patient activities. However, the increase in accuracy was not consistent across all new data additions, suggesting a complex relationship between the amount of data and the performance of the models. The complete details of this analysis, including the methodology, data processing, and a full discussion of the results, are presented in the Additional file 1 under Section S1.

### Domain adaptation

In this set of experiments, the DANN domain adaptation method was applied to the healthy-to-healthy and healthy-to-PwPD cases before cross-testing. No labels from the test data were used with the domain adaptation method. We use a separate source (training) and target (testing) dataset when using a DANN. We have a healthy source dataset (PAMAP2 or MHEALTH or both combined) and a target data (PD data in this case). The augmented source data with reoriented target data were used for these tests since it consistently improved results in the previous experiments. After normalization, the best-performing model from the source dataset’s cross-validation was loaded and applied directly to the target dataset.

DANN was first applied to the healthy-to-healthy test cases. The average accuracy results when PAMAP2 and MHEALTH were used as the source data for cross-testing are presented in Table 5. These results show that cross-testing with domain adaptation resulted in the best accuracy of 88.37% for PAMAP2 as the source and 76.01% for MHEALTH as the source, using Chen [10] and Jordao [16] architectures. These results were comparable to those obtained without domain adaptation, indicating that domain adaptation did not significantly improve generalizability. Moreover, the average accuracy for cross-testing across all architectures was higher than that with DANN for both cases where the source dataset is PAMAP2 and MHEALTH, and the target is the opposite. Although DANN did not perform as expected, the HAR literature consistently aligns with domain adaptation results. Furthermore, while DANN performed better than cross-testing in some cases, their architectures consistently misclassified some labels.

**Table 5:** The average cross-testing accuracy and F1 scores, in parentheses, with domain adaptation for PAMAP2 and MHEALTH

	Base Model	O’Halloran [13]	Wan [14]	Burns [15]	Chen/Jordao [10, 16]	Kalouris [17]	Yatbaz [18]	Rueda [19]
$$\begin{array}{l}\hbox {Source: P2 Aug} \\ \hbox {Target: MH Reorient}\end{array}$$	$$\begin{array}{l}14.17\\ (3.52)\end{array}$$	$$\begin{array}{l}77.39\\ (76.68)\end{array}$$	$$\begin{array}{l}43.15\\ (79.50)\end{array}$$	$$\begin{array}{l}80.21\\ (79.70)\end{array}$$	$$\begin{array}{l} 88.37\\ (86.85)\end{array}$$	$$\begin{array}{l}80.72\\ (80.89)\end{array}$$	$$\begin{array}{l}73.50\\ (73.03)\end{array}$$	$$\begin{array}{l}70.50\\ (69.96)\end{array}$$
$$\begin{array}{l}\hbox {Source: MH Aug} \\ \hbox {Target: P2 Reorient}\end{array}$$	$$\begin{array}{l}49.55\\ (40.21)\end{array}$$	$$\begin{array}{l}63.01\\ (62.46)\end{array}$$	$$\begin{array}{l}63.17\\ (54.15)\end{array}$$	$$\begin{array}{l}75.96\\ (76.05)\end{array}$$	$$\begin{array}{l} 76.01\\ (74.27)\end{array}$$	$$\begin{array}{l}77.12\\ (75.34)\end{array}$$	$$\begin{array}{l}64.82\\ (64.80)\end{array}$$	$$\begin{array}{l}15.81\\ (4.32)\end{array}$$

The data augmentation notation applies to the MHEALTH and PAMAP2 source datasets. ‘PD Reorient’ indicates PD data with sensor axes reoriented to match the source data

**Fig. 3:**
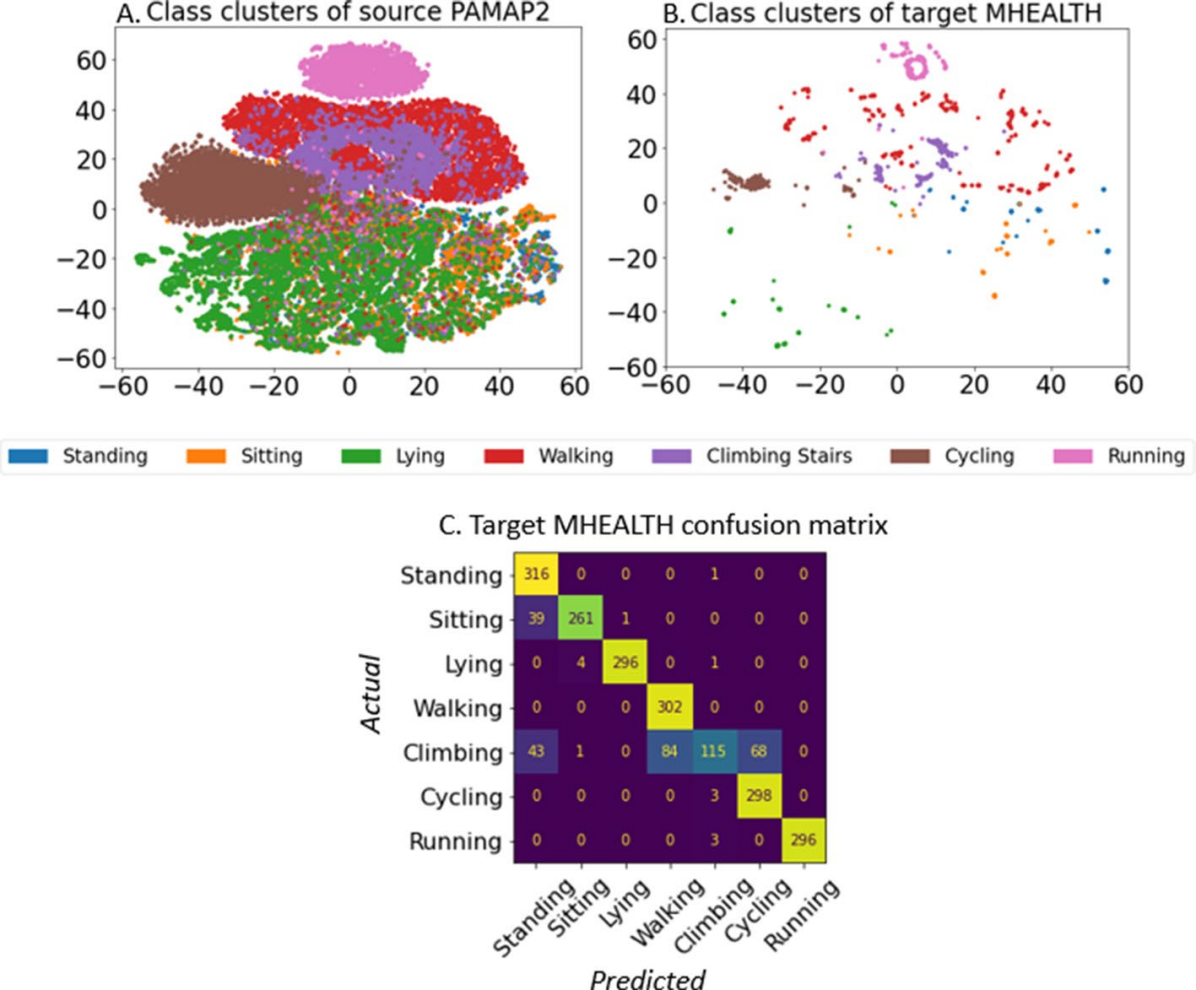
tSNE plots with DANN applied for the PAMAP2-to-MHEALTH test case using Chen/Jordao architecture [10, 16]. **A** Augmented PAMAP2 as the source data, and **B** reoriented MHEALTH as the target data. **C** Confusion matrix on target MHEALTH, where the labels represent classes: standing, sitting, lying, walking, climbing stairs, cycling, and running

Figure 3 shows the tSNE plots of the Chen/Jordao model’s architecture [10, 16] for DANN, with PAMAP2 as the source and MHEALTH as the target. The figure demonstrates that DANN aligned the domain spaces between the source and target. However, the blue, orange, and green dots overlap in the center, indicating that the model had difficulty distinguishing between standing, sitting, and lying down during training. The red and purple dots also overlap in Fig. 3A, indicating that the walking and climbing stairs activities may have also been indistinguishable. The classes in the target MHEALTH data tSNE plot (Fig. 3B) are well-separated, but there is a similar overlap between walking and climbing stairs, as well as an overlap between sitting and standing, indicating the challenge of accurately classifying these activities. The confusion matrix for this test case is included in Fig. 3C for better visualization. It shows that the model had difficulty predicting some activities, such as sitting, standing, and climbing stairs, while performing well for the others.

The domain adaptation techniques were then tested on the healthy-to-PwPD case for each architecture. The binary F1 score and accuracy results are presented in Table 6. When PAMAP2 was used as the source, domain adaptation performed better in five out of eight tests than cross-testing without DANN. Furthermore, domain adaptation was more effective for more complex models, achieving F1 scores in the mid-high 40% range. When MHEALTH was used as the source, domain adaptation performed slightly better for more complex models. Notably, models trained on PAMAP2 performed better than models trained on MHEALTH when applied to the PD dataset. This can be because the PAMAP2 dataset is much larger, particularly with data augmentation, which provides more training samples for the models to successfully classify the PD dataset.

**Table 6:** PAMAP2 and MHEALTH to PD cross-testing binary accuracy and F1 Scores with Domain Adaptation

	Base Model	O’Halloran [13]	Wan [14]	Burns [15]	Chen/Jordao [10, 16]	Kalouris [17]	Yatbaz [18]	Rueda [19]
$$\begin{array}{l}\hbox {Source: P2 Aug} \\ \hbox {Target: PD Reorient}\end{array}$$	$$\begin{array}{l}40.01\\ (6.06)\end{array}$$	$$\begin{array}{l}62.45\\ (21.43)\end{array}$$	$$\begin{array}{l}64.83\\ (43.65)\end{array}$$	$$\begin{array}{l}66.67\\ (44.62)\end{array}$$	$$\begin{array}{l}70.19\\ (45.18)\end{array}$$	$$\begin{array}{l}73.02\\ (49.79)\end{array}$$	$$\begin{array}{l}69.79\\ (44.08)\end{array}$$	$$\begin{array}{l}67.02\\ (0.04)\end{array}$$
$$\begin{array}{l}\hbox {Source: MH Aug} \\ \hbox {Target: PD Reorient}\end{array}$$	$$\begin{array}{l}61.89\\ (19.99)\end{array}$$	$$\begin{array}{l}69.10\\ (37.49)\end{array}$$	$$\begin{array}{l}63.35\\ (24.13)\end{array}$$	$$\begin{array}{l}67.49\\ (20.16)\end{array}$$	$$\begin{array}{l}57.00\\ (19.58)\end{array}$$	$$\begin{array}{l}62.80\\ (29.35)\end{array}$$	$$\begin{array}{l}66.23\\ (34.01)\end{array}$$	$$\begin{array}{l}68.09\\ (3.97)\end{array}$$

The average values are shown, with F1 scores presented in parentheses. The notation for data augmentation applies to both the MHEALTH and PAMAP2 datasets. ‘PD Reorient’ indicates PD data with sensor axes reoriented to match the source data

**Fig. 4:**
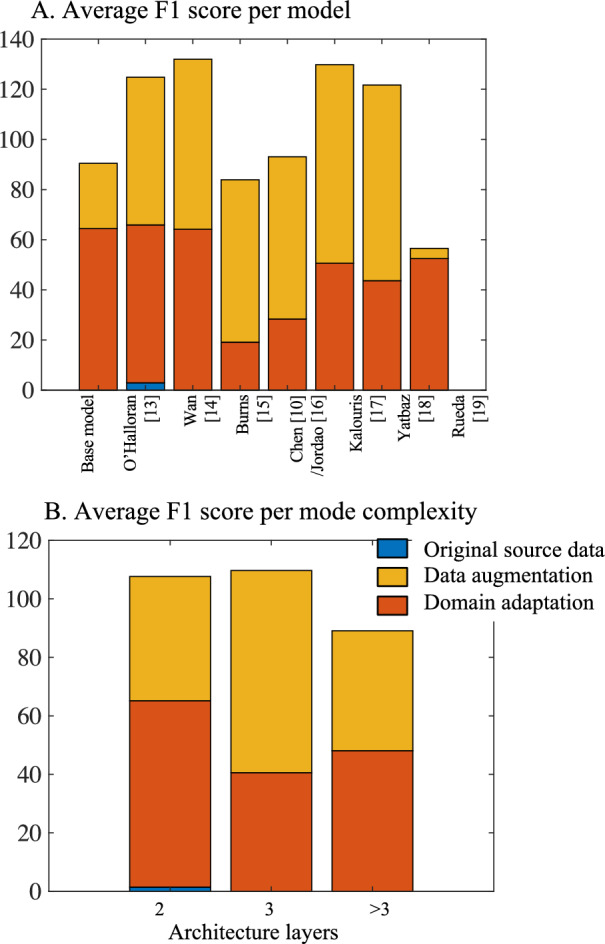
Average F1 scores for cross-testing PAMAP2 and MHEALTH to PD dataset with **A** different architectures and **B** different model complexities

The average F1 score of cross-testing of PAMAP2 and MHEALTH individually on the PD dataset for each architecture is shown in Fig. 4. The figure also shows the results for architecture models with different complexities, as indicated in Fig. 1A. Overall, data augmentation and domain adaptation improved the models’ generalizability from healthy to diseased populations. Lower complexity architectures, such as the base model and those in O’Halloran [13] and Wan [14], benefited most from data augmentation, while mid-level complexity architectures benefited more from domain adaptation. Surprisingly, the most complex model in [19] benefited the least from domain adaptation.

An additional experiment was conducted to explore whether combining the MHEALTH and PAMAP2 augmented datasets into one large dataset for training, similar to our prior cross-testing experiment, would improve results when testing the model on the PD dataset with domain adaptation. We ran this test solely utilizing the Wan model [14] because it performed best without domain adaptation, as was observed in Table 4. This experiment yielded an accuracy of 64.17% and an F1 score of 21.00%. These results are surprisingly lower than those obtained without domain adaptation in Table 4 and those using a single dataset as the source seen in Table 6.

In another experiment, we assessed the impact of the domain adaptation when progressively introducing PD patient data into the training set. We incrementally added two PD subject’s data to the healthy training data at a time, and evaluated the model performance. This was specifically to evaluate whether the pretraining of models with healthy subject data (source domain) followed by the addition of PD data (target domain) would improve the model’s performance in recognizing PD-specific activities. The results from this experiment demonstrated that the accuracy of the models did improve as PD patient data were incrementally added to the training set, illustrating the benefits of domain adaptation. However, the improvement in accuracy reached a plateau, indicating that there is a limit to how much the addition of target domain data can enhance the model’s performance. A more detailed analysis of this experiment is provided in Additional file 1 under Section S2.

## Discussion

This study addresses the challenge of developing accurate classification models for diseased and less-represented populations in wearable data, which limits the application of health and home monitoring systems in clinical decision-making. To address this challenge, the study created a simple, shallow base model and implemented various regularization techniques to provide a more robust and effective model for activity recognition in PwPD. The study also compared the proposed model’s performance with existing CNN architectures from the literature to provide insights into the impact of model complexity on activity recognition performance. In addition, the study implemented the state-of-the-art domain adaptation technique, DANN, to explore its effectiveness in improving the activity classification capabilities of deep NN models. These contributions provide valuable insights for future research and contribute to advancing the field of human activity recognition.

First, across almost every architecture on the PAMAP2 and MHEALTH datasets, the average cross-validation accuracy increased when training on augmented data compared to the original data. This trend was especially prominent, with more complex models adding more layers to the architecture. The only architecture where this trend was not observed was the base model. This suggests that enhancing a dataset with data augmentation justifies using a more complex model with a significantly increased dataset size. The data augmentation also helped alleviate the issue of subject variability in the activity recognition data. Single subjects were held out for testing across all architectures, leaving the models vulnerable to the variability each person exhibits in how they perform common activities of daily living. A wide range of accuracy values was observed in the cross-validation folds before applying data augmentation, particularly in fold eight of the PAMAP2 dataset, where subject 8 was the only left-handed subject with sensors on the left-side body. In the original data experiments, fold eight consistently displayed lower accuracy when held out for testing. With data augmentation applied, each cross-validation fold had accuracy scores significantly closer in value, including the left-handed subject 8 of PAMAP2 tested against all right-handed sensor data. The low accuracy score with PAMAP2 subject 8 was only alleviated with data augmentation. The main reason is that some left-hand movements reflect the right-hand movements, which means moving in the opposite direction. The model trained on augmented data captured the activity’s patterns independent of the sensor placement on the left or right wrist.

For the healthy-to-PwPD test case scenario, there is a clear advantage to incorporating data augmentation in the source model. There was also a significant impact on model complexity regarding the binary F1 score for the updated three unique activity labels in the PD data. Table 4 clearly shows that by cross-testing from MHEALTH or PAMAP2 and applying the model to the PD dataset, the F1 score is higher when the model is less complex. This observation does not depend on the dataset used for training since it was observed for both MHEALTH and PAMAP2. Also, this is simultaneously observed when using a training dataset of combined augmented MHEALTH and PAMAP2. Similarly, when domain adaptation is applied, DANN performs better using models with moderate complexity (Table 6). These findings confirmed data augmentation and domain adaptation gained more benefit in the average F1 score from a healthy source to the diseased target population (Fig. 4).

Our exploration into combining augmented MHEALTH and PAMAP2 to form one large training dataset is an interesting point of discussion. It was observed that this larger training dataset allowed for simpler model architectures to perform better with higher accuracy and F1 scores in Table 4. The best result was utilizing the Wan model [14]. However, when domain adaptation was involved, our experiment with the Wan model [14] yielded unexpectedly worse results. In our future work, we plan to dive deeper into this exploration and investigate the reason for poor domain adaptation performance when trained on two datasets combined.

Comparing the results from the healthy-to-healthy tests to the healthy-to-PwPD tests is also interesting to note. The domain adaptation methods seemed to improve model performance more in the healthy-to-PwPD case than healthy-to-healthy. Binary F1 scores for the healthy-to-PwPD tend to be low because of the large domain shift between the healthy and diseased datasets. The highest average F1 score was 49.79% when training on PAMAP2 and testing on PD with DANN applied. Another factor involved is the datasets. The MHEALTH dataset is much smaller than PAMAP2, and the PwPD dataset is much smaller than both MHEALTH and PAMAP2, especially with augmented training data. There is a large class imbalance when comparing the source to target datasets, which can lead to additional challenges in classification.

It is worth emphasizing that the lower performance observed during standing activities in Fig. 2 could be due to the involuntary movements PD patients commonly experience, known as dyskinesia. Dyskinesia can result in unpredictable, uncontrollable, and involuntary movements that cause difficulty in accurately classifying standing activities. These movements can make it challenging for individuals with PD to maintain a stable posture, leading to difficulties differentiating between standing and other activities. In addition, within the parameters of this study, it is difficult to say definitively if and how the age or gender of the populations compared would impact performance.

The only work close to ours in cross-testing from healthy to PwPD is the work of Albert et al. [4]. They used smartphone accelerometer data and achieved an accuracy of 63.5%, which is significantly lower than the accuracy of 73.02% that we achieved by adapting the PAMAP2 data to PwPD data using Kalouris et al. [7] (Table 6). Hysseini et al. [11] conducted a similar work by classifying activities and cross-testing but from adult to children activity dataset. They achieved higher binary F1 scores than our healthy-to-PwPD cross-testing cases, with 70% for standing, 63% for walking, and 52% for sitting. This suggests a larger domain shift between healthy and diseased populations, specifically PwPD, who experience a much greater shift in their movement patterns. The present study underscores the need for further research to develop accurate models to improve clinical decision-making in PD patients. Such models should be designed to account for the larger domain shift and other challenges associated with classifying activities in diseased populations.

Regarding study limitations, it is acknowledged that the PD dataset used in this study is relatively small compared to some studies involving healthy subjects [1]. However, it should be noted that the dataset includes 14 subjects, which is comparable in size to other PD datasets used in previous studies [4–6]. Furthermore, the PD dataset was only used for testing and not for training—except for an additional experiment where it was combined with data from healthy subjects. Consequently, the size of the dataset did not impact the generalizability of the models from the healthy to the PwPD dataset. The PD data also did not have activity labels that matched those in the MHEALTH and PAMAP2 datasets, which presents variability in the activities-matching process between datasets. However, activities were mapped to their closest analogs. It is also important to note that each scenario, based on its unique dataset, sensor placement, and instruction method, introduces nuances that may affect the outcomes. Our findings, though based on different datasets and varying numbers of activities, aim to offer a comprehensive perspective. It underscores the importance of viewing our conclusions in the broader human activity recognition research context, emphasizing the continuous evolution and refining of methodologies in this field. While our study could not perform PD-to-PD cross-testing due to the absence of a suitable public dataset, this limitation highlights the importance of our work. There is a clear need for advanced algorithms in human activity recognition, especially for disease populations like Parkinson’s, which differ significantly from the general population. Our work points to the urgency and potential for future research in this vital area.

Despite these limitations, the contribution of this study lies in the pioneering exploration of domain adaptation and data augmentation techniques for healthy-to-PwPD cross-testing in activity recognition. The findings of this study have the potential to inspire future research in this area and guide the development of more accurate and robust deep NN models for human activity recognition in PwPD.

## Conclusions

This study explored the potential of deep NN models for activity recognition in PwPD using wearable sensor data. Several experiments were conducted using datasets from PAMAP2 and MHEALTH, focusing on the generalizability of the models from healthy, young populations to PwPD. The effects of data augmentation, manual axis reorientation, model complexities, and domain adaptation were thoroughly examined. The results showed that the best activity recognition performance was achieved by a moderately complex model trained on the augmented PAMAP2 dataset and adapted to the PD domain using the DANN domain adaptation method. The F1 score of this model was 49.79%, and the accuracy was 73.02%, significantly higher than the accuracy of 63% reported in previous studies.

Several key findings were produced from this study. First, it was discovered that more complex models did not always perform better when trained on healthy data and applied to the PwPD domain, despite data augmentation and domain adaptation techniques. Second, it was found that lower complexity models benefitted the most from data augmentation on healthy models for the disease population domain. Last, domain adaptation was shown to improve the generalizability of the models, with the most significant improvements observed for models with moderate complexity levels. These findings can potentially contribute to developing accurate wearable-based activity monitoring solutions for PwPD, improving clinical decision-making based on patient activity levels.

To improve the accuracy of activity recognition models for PwPD, future research should focus on including more relevant activities in the training data and incorporating PwPD data in the training process to improve model performance. Further research should be conducted to evaluate model performance when utilizing larger training datasets, which can be accomplished by combining datasets. For future work, we plan to investigate the reason for poor domain adaptation performance for some models, especially when trained on two datasets.

## Methods

### Datasets

**Fig. 5:**
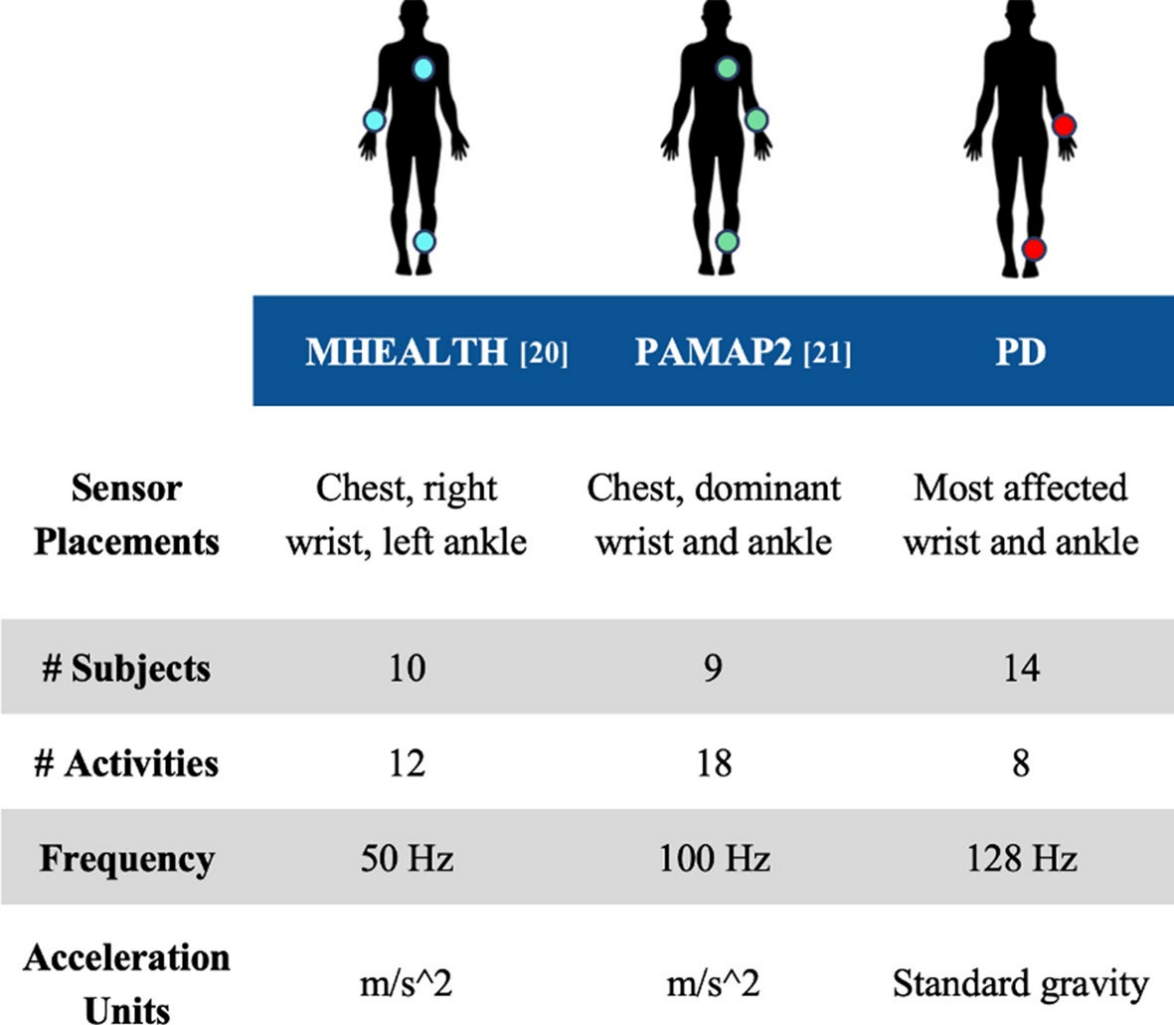
The figure compares the datasets used in this study

**Fig. 6:**
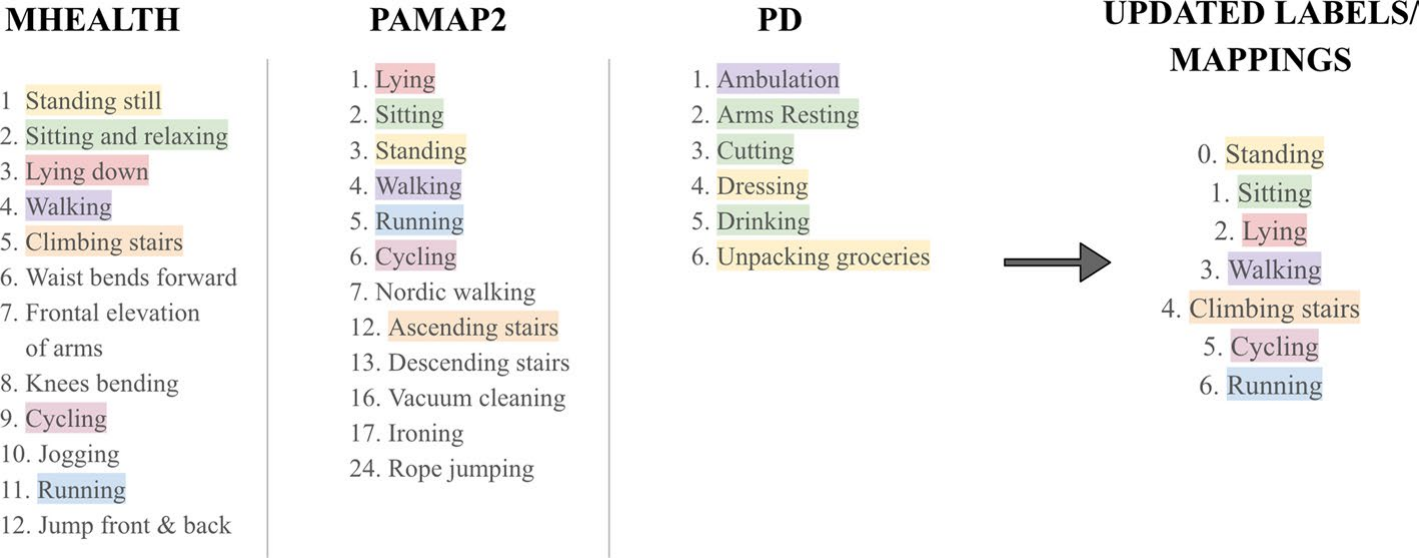
The activity labels across the MHEALTH, PAMAP2, and PD datasets, with matching activities indicated by the same color

The study utilized three datasets, namely two publicly available datasets—MHEALTH [20] and PAMAP2 [21]—along with a privately collected dataset of PwPD, referred to as PD data. The datasets contained wearable motion sensor readings on common activities of daily living. The collected readings were from two accelerometer sensors mounted on the wrist and ankle. Some of the main activities were standing, sitting, laying, and walking (refer to Fig. 6 for a full list of activities in each of the datasets). A summary of the characteristics and comparisons between these datasets is presented in Fig. 5.

- MHEALTH: The MHEALTH dataset consisted of 10 participants performing 12 activities while wearing an IMU sensor on their right-side wrist, left-side ankle, and chest. Each activity had an average duration of 1 min per subject.
- PAMAP2: The PAMAP2 dataset consisted of 1 female and 8 male participants with an average age of 27 ± 3 years, who performed 18 activities with an IMU sensor on their dominant-side wrist and ankle and a chest IMU sensor. Each activity had an average duration of 2.8 min per subject.
- PD: The PD dataset used in this study was obtained from a previous research study [23, 24], and the authors of this paper were granted access to the data for analysis purposes. The patients gave informed consent, and the University of Rochester and Great Lakes Neurotechnologies institutional review boards approved the data collection procedure. Fifteen PD subjects (6 F, 9 M) with an average age of 58 ± 10 years participated in this study. The subjects had an average disease duration of 10±4, a total mAIMS of 5 ± 4.4, an average 26-item PD dyskinesia Scale (PDYS-26) of 35 ± 21, and an equivalent daily dose of levodopa 1226 ± 535. The subjects performed six activities while wearing an accelerometer on the wrist and ankle of their most affected side. These activities included walking, sitting on a chair, using a knife and fork to cut food while standing, putting on and taking off a coat while standing, drinking water from a cup while sitting, and unpacking groceries while standing. These activities were repeated four rounds during the patient’s medication ON and OFF times, providing a range of disease manifestations to assess the generalizability of deep NN models to PwPD. The subjects were asked to stop their PD medications the night before the experiment, so they performed the first round of experiments in their medication OFF time. After performing the first round, the subjects resumed their normal PD medications. A neurologist identified when the medication kicked in and then asked the subjects to perform the second round of the experiment. The other two rounds were 1 h apart. The 4-h procedure allowed for changes in PD symptoms to be captured during subjects’ medication ON and OFF times. The data duration ranged from 12 to 16 min for each subject, with an average time of 14 min.

### Data preparation

During the data preparation phase, the publicly available datasets were standardized to align with the setup of the PD dataset.

*Matching sensor placement* To match the sensor placement across all three datasets, the following steps were taken:

- The chest sensor data were removed from the MHEALTH and PAMAP2 datasets, leaving only the wrist and ankle sensor data, also in the PD dataset. The use of two sensors instead of one has many advantages, including higher performance, reduced uncertainty, and increased confidence [25, 26].
- The gyroscope and magnetometer data were removed from the MHEALTH and PAMAP2 datasets so that only accelerometer data were retained across all three datasets.
- Data labeled as ‘null class’ indicating transient or unspecified activities were removed from the MHEALTH and PAMAP2 datasets.
- The PAMAP2 dataset had some samples with missing or NaN values. These samples were removed from the edited dataset used for analysis.

*Matching units between datasets* To match the units between the datasets, the following steps were taken:

- The PAMAP2 (100 Hz) and PD (128 Hz) datasets were down-sampled to match the MHEALTH sampling frequency of 50 Hz.
- The acceleration unit in the PD dataset was converted to $m/s^2$, the same as the units used in the PAMAP2 and MHEALTH datasets.

*Data segmentation* The following steps were taken to ensure that the data were appropriately segmented for analysis:

- The data were segmented into windows of 150 timestamps, which correspond to 3 s with a 50 Hz sampling frequency.
- As recommended by [27], 5 s of data from the beginning and end of each labeled activity in the MHEALTH and PAMAP2 datasets were removed to eliminate any transient data from the recordings.

*Matching activities* To match the activities across the datasets, the following steps were taken:

- Seven common activities between PAMAP2 and MHEALTH, namely ‘Standing,’ ‘Sitting,’ ‘Laying,’ ‘Walking,’ ‘Climbing Stairs,’ ‘Cycling,’ and ‘Running’ were analyzed.
- PAMAP2 subject nine was removed from the analysis as they did not perform the above activities.
- The PD activities were mapped to the closest analogs of those seven activities in MHEALTH and PAMAP2. In specific, Ambulation was mapped to Walking, Arms Resting to Sitting, Cutting to Sitting, Dressing to Standing, Drinking to Sitting and Unpacking Groceries to Sitting.

*Data normalization* Finally, all the data were normalized before segmentation by performing the following steps:

- The mean and standard deviation of each column/axis in the training data were obtained.
- The mean was subtracted from each value, and the result was divided by the standard deviation.
- The same normalization was applied to the testing data, with the mean and standard deviation calculated separately from the training data.

These steps ensured that the data were appropriately standardized before any further analysis. The original and updated activities in MHEALTH and PAMAP2 and the activity mapping in the PD data are depicted in Fig. 6. After matching activities across datasets, the MHEALTH dataset consisted of 2132 samples, each with 150 timestamps and 6 features for each timestamp. The PAMAP2 dataset consisted of 5,748 samples, each with 150 timestamps and 6 features for each timestamp. For each dataset, the 6 features were the raw 3D accelerometer readings from the wrist and ankle sensors instead of the resultant acceleration. Using the resultant acceleration leads to losing the angular acceleration, which is helpful in distinguishing between activities.

### Data augmentation

Following the methodology proposed in [8], three data augmentation techniques were employed on the MHEALTH and PAMAP2 datasets. The code for these methods is publicly available from the authors of [8]. The augmentation techniques used in this study are briefly described as follows:

- “Rotation” arbitrarily rotates the axes of a window segment to simulate different sensor placements and orientations. The manual reorientation described next considered significant changes in the orientation, such as 180 degrees and sensor placement, that changed the order of the axes between the datasets. However, sensor rotation as a data augmentation was limited to -90 to 90 degrees of change. This level of augmentation was chosen to prevent the model from overfitting and to make the training possible at the same time by not corrupting the activity patterns.
- “Jitter” introduces Gaussian noise sampled with a standard deviation of 0.05 to mimic potential sensor noise.
- “Scaling” multiplies the data in a window by a random scalar sampled from a normal distribution with a standard deviation of 0.1 to mimic multiplicative noise.

The latter two methods are specifically designed to introduce variations in signal data commonly found in Parkinson’s data due to motor complications of the condition, such as tremors. For each window of data in the MHEALTH and PAMAP2 datasets, eight new data samples were generated. Each augmented sample had rotation, jitter, and scaling augmentations combined. It is important to note that this process can be computationally expensive.

After the data augmentation step, The MHEALTH dataset consisted of 19,188 samples, each with 150 timestamps and 6 features for each timestamp. After augmentation, the PAMAP2 dataset consisted of 51,732 samples, each with 150 timestamps and 6 features for each timestamp.

### Manual reorientation

The orientation of each sensor was not explicitly reported in the dataset description. As a result, a manual reorientation process was employed in addition to data augmentation. This process involved visually analyzing the graphs of various activities to derive a mapping of one dataset’s axes to another. The standing, sitting, and walking activities were the focus since the ankle and wrist are usually in predictable positions for those activities, which allows for a more generalized mapping of the baseline orientation of the sensor axes.

The reorientation process involved multiplying all values in a column/axis by -1 if negated in the mapping and reordering the columns according to the new axes’ order. The target dataset was reoriented during cross-testing to match the source dataset the model originally trained on. It should be noted that this method may be error-prone, as the mapping can vary slightly depending on the analyzed subjects, windows, and activities. In addition, in the PAMAP2 and PD datasets, each patient’s sensors may be placed on different sides of the body, resulting in different mappings. Furthermore, if the sensor shifts or moves during data collection, the baseline orientation can appear different across the data windows. Despite these challenges, this method significantly improved cross-testing performance when combined with data augmentation compared to data augmentation alone. Matching most of a source dataset’s sensor axes’ orientation to a target dataset’s orientation is preferable to matching none.

Upon reorientation to the MHEALTH dataset, the PD dataset contained 6,539 samples, comprising 150 timestamps and 6 features for each timestamp. After reorienting the PD dataset to the PAMAP2 dataset, the PD dataset consisted of a similar number of samples. Table 7 illustrates the number of samples per activity across the datasets before and after data augmentation of MHEALTH and PAMAP2. The PD dataset was not augmented since it was a hold-out testing set.

**Table 7:** The size of each dataset

	MHEALTH		PAMAP2		PD
	Original	Augmented	Original	Augmented	
Standing	317	2853	909	8181	3042
Sitting	301	2709	979	8811	2843
Lying	301	2709	956	8604	x
Walking	302	2718	1132	10,188	654
Climbing Stairs	311	2799	535	4815	x
Cycling	301	2709	784	7056	x
Running	299	2691	453	4077	x
Total	2132	19,188	5748	51,732	6539

It includes the number of samples for each activity for original and augmented datasets. For PD, MH R indicates the PD data reoriented to MHEALTH, and P2 R indicates PD reoriented to PAMAP2

### Convolutional neural network (CNN) architectures

**Fig. 7:**
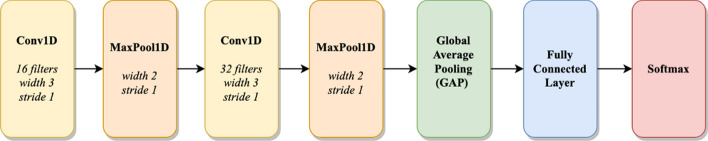
The figure depicts the architecture of the proposed base CNN model

In the field of human activity recognition, overfitting is a prevalent issue in models trained on smaller datasets. A simple, shallow base model was designed to mitigate this problem, incorporating various regularization techniques to minimize overfitting within and across domains. As shown in Fig. 7, the proposed architecture consists of a 1D convolutional layer with 16 kernels of width three and stride of one, followed by a 1D max pooling layer with width two and stride of one. An additional 1D Conv/1D Max pool block with 32 kernels is added, leading to global average pooling, a fully connected layer with 50% dropout, and final Softmax layers. The model was trained for 60 epochs with a batch size of 64, a learning rate of 0.001, Adam optimizer, categorical cross-entropy loss, and L1/L2 regularization with a value of 0.001 applied at every convolutional layer. The hidden layers utilized the ReLU activation function, whereas Softmax was used for the final output layer. The total number of parameters in the base model was 2103.

To assess the impact of model complexity on testing performance, several CNN architectures with varying complexity developed on the MHEALTH and/or PAMAP2 datasets were replicated as closely as possible following the descriptions from the literature [10, 13–19]. These models were implemented using Keras and trained on Florida Atlantic University’s High-Performance Computing (HPC) Cluster for remote GPU access. For some replicated models, a flattening or pooling layer was not specified before the fully connected layers preceding the output of the CNN and the convolutional blocks. As a result, a single flattening layer was included in these models to ensure compatibility within the architectures. Adam optimizer, categorical cross-entropy loss, and ReLU activation function were used for papers that did not clarify the optimizer, loss function, or activation function type [10, 14–17, 19], to maintain consistency with the majority of the other papers and the proposed base model. To implement the work by Chen et al. [10], the approach by Jordao et al. [16] was followed, as the learning rate was not specified. A learning rate of 0.001 was used to allow the model to converge. In work by O’Halloran et al. [13], the filter sizes for the convolutional and max pooling layers were not specified. However, since this architecture had two convolutional and max pooling blocks, similar to the base model, the pooling and convolutional filters’ width and stride were kept similar to the proposed model. To prevent it from being too similar to the base model structure, 16 and 16 filters were used instead of 16 and 3. Figure 1 shows the complexity of the implemented models in terms of the number of layers and parameters. The colors in Fig. 1A indicate the order of complexity, the same order of which is maintained throughout the tables.

### Domain adversarial neural network (DANN)

The Domain Adversarial Neural Network (DANN) [9] was implemented in this study to investigate its potential to improve the deep NN models’ activity classification capabilities. DANN is a state-of-the-art adversarial domain adaptation technique commonly used in domain classification problems and HAR. The Awesome Domain Adaptation Python Toolbox (ADAPT) package [28] was used for this portion. DANN is a feature-based domain adaptation technique commonly used in domain classification problems and HAR by aligning the distributions of features across different domains through standard backpropagation training. The DANN architecture comprises a feature extractor, label predictor, and domain classification. During training, the label predictor predicts class labels, and simultaneously, the domain classifier differentiates between the source and target domains. The goal is to learn features that are both discriminative and domain-invariant so that the model can classify input data into different classes, while the source and target data cannot be differentiated and thus will be treated similarly.

The DANN feature extractor was the final dense layer of each CNN architecture used in this study. The label predictor consists of a 50% dropout layer followed by a dense layer with Softmax activation, and the domain classifier consists of a dense layer with Elu activation followed by another dense layer with Sigmoid activation. The DANN was compiled with Adam optimizer with a learning rate 0.01 and Mean Squared Error loss function. The DANN was trained with a lambda value of 0.1 and a batch size of 32 for 100–350 epochs, depending on when the model converged. The same optimizer used in the CNN architecture that makes up the feature extractor was used in this training stage.

### Supplementary Information

**Additional file 1.** **Figure S1.** The learning curve illustrates the impact of incrementally adding healthy subject data to the training set on model accuracy when tested against PD data. The initial model was trained using augmented data from a combination of PAMAP2 and MHEALTH datasets. With each additional subject's data incorporated (one from each dataset), there is a general trend of improved accuracy, albeit with notable variations. This pattern highlights the complex relationship between training data volume and model performance in the context of human activity recognition for PD. **Figure S2.** The curve presents the effects of systematically introducing PD subject data into the training process using the Domain Adaption Neural Network (DANN) approach, with the Kalouris model and PAMAP2 healthy dataset as the source. The figure displays the progression in model accuracy as more PD subjects are added, indicating the benefits of source domain pretraining. It also reflects a plateau, suggesting a point of diminishing returns in model performance improvements, which provides important insights into the optimization of transfer learning strategies for PD activity recognition.

## Data Availability

The PAMAP2 and MHEALTH activity datasets used in this study are publicly accessible. In addition, the PD dataset utilized and analyzed in this research can be obtained from the corresponding author upon reasonable request.

## Abbreviations

NN: Neural network
PwPD: Patients with Parkinson’s disease
PD: Parkinson’s disease
HAR: Human activity recognition
CNN: Convolutional neural network
DANN: Domain adversarial neural network
IMU: Inertial measurement unit
PDYS-26: PD dyskinesia scale
ADAPT: Awesome Domain Adaptation Python Toolbox
