# Supplementary material for: Deep neural networks for wearable sensor-based activity recognition in Parkinson’s disease: investigating generalizability and model complexity
Source: Biomed Eng Online. 2024 Feb 9;23:17. doi: 10.1186/s12938-024-01214-2 (PMC10858599; doi:10.1186/s12938-024-01214-2)
Supplement: Supplementary file 1 — Additional file 1. Figure S1. The learning curve illustrates the impact of incrementally adding healthy subject data to the training set on model accuracy when tested against PD data. The initial model was trained using augmented data from a combination of PAMAP2 and MHEALTH datasets. With each additional subject's data incorporated (one from each dataset), there is a general trend of improved accuracy, albeit with notable variations. This pattern highlights the complex relationship between training data volume and model performance in the context of human activity recognition for PD. Figure S2. The curve presents the effects of systematically introducing PD subject data into the training process using the Domain Adaption Neural Network (DANN) approach, with the Kalouris model and PAMAP2 healthy dataset as the source. The figure displays the progression in model accuracy as more PD subjects are added, indicating the benefits of source domain pretraining. It also reflects a plateau, suggesting a point of diminishing returns in model performance improvements, which provides important insights into the optimization of transfer learning strategies for PD activity recognition. [file 12938_2024_1214_MOESM1_ESM.docx]

**Additional file**

Deep Neural Networks for Wearable
Sensor-Based Activity Recognition in Parkinson’s
Disease: Investigating Generalizability and Model
Complexity

**Section S1. Incremental Data Integration for Model Training**

In this experiment, we initiated training with a base dataset composed of augmented data from three healthy subjects from the PAMAP2 dataset and two from the MHEALTH dataset. Subsequent models were then incrementally trained with additional data from one more subject from each dataset. We took care to exclude the validation subject from PAMAP2 in all training sets to maintain the integrity of our evaluation. As we reached the limits of the PAMAP2 dataset, we incorporated data from an additional two subjects from the larger MHEALTH dataset, beginning with the sixth model iteration. To ensure consistency, all data, including that from PD subjects, were reoriented to align with the PAMAP2 sensor orientation.

As shown in Figure S1, the accuracy generally increased as more healthy subject data were added, which underscores the potential benefits of a larger source dataset. However, the learning curve also reveals fluctuations that suggest a complex relationship between additional data and model performance, possibly hinting at the heterogeneity of the healthy data or the limitations of the models in capturing the variance within PD data.


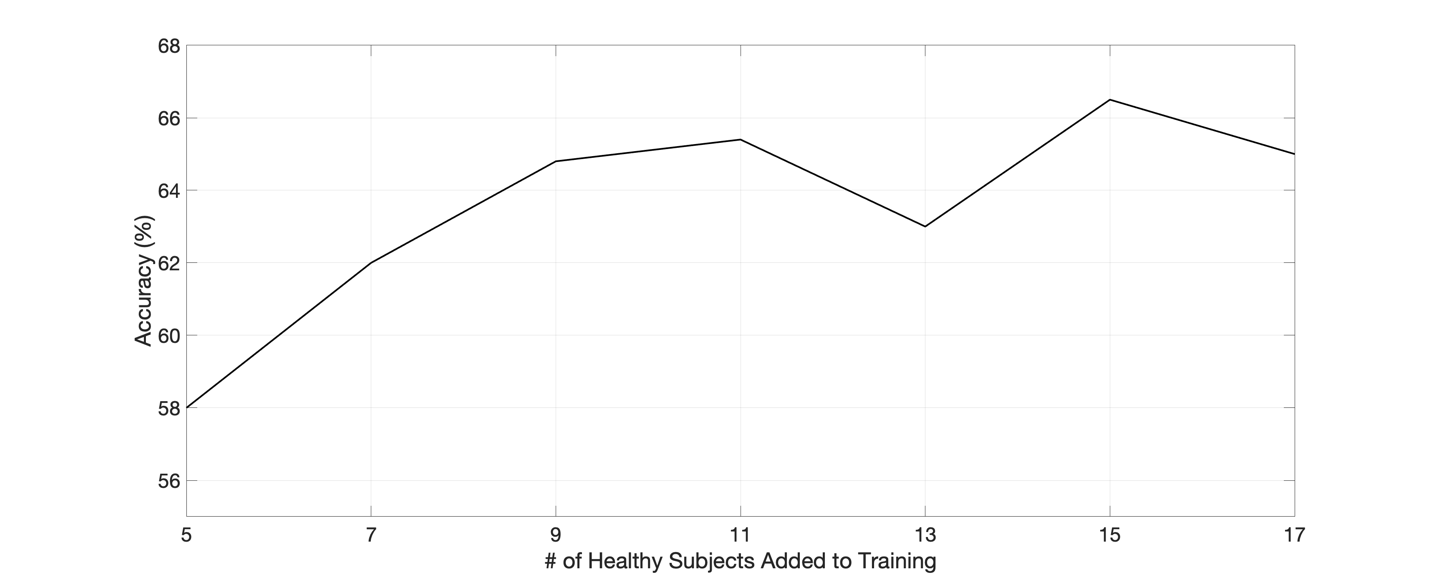


**Figure S1 –** The learning curve illustrates the impact of incrementally adding healthy subject data to the training set on model accuracy when tested against PD data. The initial model was trained using augmented data from a combination of PAMAP2 and MHEALTH datasets. With each additional subject's data incorporated (one from each dataset), there is a general trend of improved accuracy, albeit with notable variations. This pattern highlights the complex relationship between training data volume and model performance in the context of human activity recognition for PD.

**Section S2.** **Evaluating Domain Adaptation with Progressive PD Data Inclusion**

In the second experiment, we used the domain adaptation method (DANN) with the Kalouris model and the PAMAP2 healthy dataset as the source, which previously demonstrated superior performance. By systematically incorporating an increasing number of PD subjects into the training data, we aimed to observe the benefits of pretraining with the source domain. The resulting trend, as depicted in Figure S2, illustrates improvements in accuracy but also presents a point of diminishing returns. This pattern is insightful, as it indicates the potential value of pretraining with source data and the incremental addition of target domain samples.

Both experiments highlight the complexity inherent in applying transfer learning strategies to human activity recognition. The results affirm the potential benefits of larger datasets and domain adaptation but also emphasize the non-linear and non-monotonic relationships that can emerge due to factors like data quality, subject variability, and model architecture limitations.


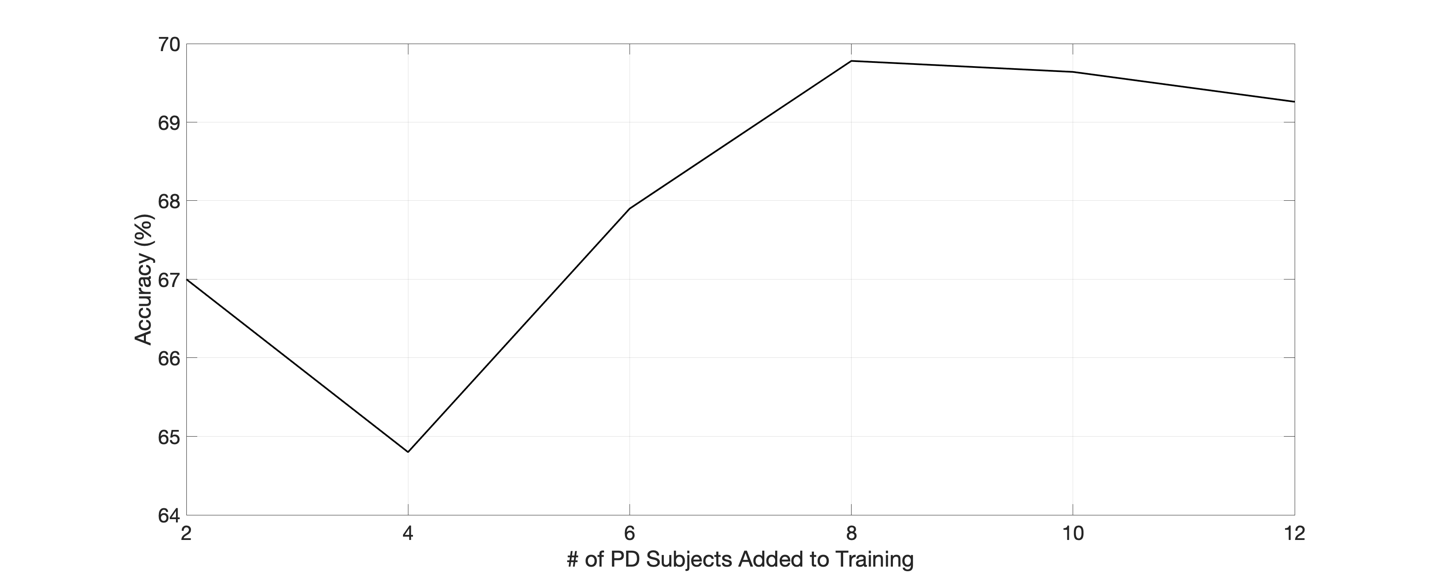


**Figure S2 –** The curve presents the effects of systematically introducing PD subject data into the training process using the Domain Adaption Neural Network (DANN) approach, with the Kalouris model and PAMAP2 healthy dataset as the source. The figure displays the progression in model accuracy as more PD subjects are added, indicating the benefits of source domain pretraining. It also reflects a plateau, suggesting a point of diminishing returns in model performance improvements, which provides important insights into the optimization of transfer learning strategies for PD activity recognition.
